# Clustered DNA Damage Patterns after Proton Therapy Beam Irradiation Using Plasmid DNA

**DOI:** 10.3390/ijms232415606

**Published:** 2022-12-09

**Authors:** Maria P. Souli, Zacharenia Nikitaki, Monika Puchalska, Kateřina Pachnerová Brabcová, Ellas Spyratou, Panagiotis Kote, Efstathios P. Efstathopoulos, Megumi Hada, Alexandros G. Georgakilas, Lembit Sihver

**Affiliations:** 1 Atominstitut, Technische Universität Wien, 1020 Vienna, Austria; 2 DNA Damage Laboratory, Physics Department, School of Applied Mathematical and Physical Sciences, National Technical University of Athens, 15780 Athens, Greece; 3 Nuclear Physics Institute, Czech Academy of Sciences, Na Truhlářce 39/64, 180 86 Prague, Czech Republic; 4 2nd Department of Radiology, Medical School, National and Kapodistrian University of Athens, 11517 Athens, Greece; 5 Radiation Institute for Science & Engineering, Prairie View A&M University, Prairie View, TX 77446, USA

**Keywords:** proton therapy beam, clustered DNA damage, linear energy transfer (LET), Agarose Gel Electrophoresis (AGE), Atomic Force Microscopy (AFM), damage biomarkers, scavenging capacity, biodosimetry

## Abstract

Modeling ionizing radiation interaction with biological matter is a major scientific challenge, especially for protons that are nowadays widely used in cancer treatment. That presupposes a sound understanding of the mechanisms that take place from the early events of the induction of DNA damage. Herein, we present results of irradiation-induced complex DNA damage measurements using plasmid pBR322 along a typical Proton Treatment Plan at the MedAustron proton and carbon beam therapy facility (energy 137–198 MeV and Linear Energy Transfer (LET) range 1–9 keV/μm), by means of Agarose Gel Electrophoresis and DNA fragmentation using Atomic Force Microscopy (AFM). The induction rate Mbp^−1^ Gy^−1^ for each type of damage, single strand breaks (SSBs), double-strand breaks (DSBs), base lesions and non-DSB clusters was measured after irradiations in solutions with varying scavenging capacity containing 2-amino-2-(hydroxymethyl)propane-1,3-diol (Tris) and coumarin-3-carboxylic acid (C3CA) as scavengers. Our combined results reveal the determining role of LET and Reactive Oxygen Species (ROS) in DNA fragmentation. Furthermore, AFM used to measure apparent DNA lengths provided us with insights into the role of increasing LET in the induction of highly complex DNA damage.

## 1. Introduction

The radiobiological and physical advantages of highly energetic proton beam therapy result in a ground-gaining field over several types of photon radiotherapy, with many proton treatment facilities—in operation or under construction—around the world. Proton beams transverse biological matter depositing a small amount of energy along the track with a low entrance dose but releasing most of their energy just before they stop at a well-defined space range called Bragg Peak. At the distal fall-off of this peak, the energy deposition stops rapidly. This property is employed in treatment planning systems to design different beam combinations that produce wider peaks, called Spread-Out Bragg Peaks (SOBP), with the desired geometrical characteristics of the tumor volume to be treated. This allows the irradiation of cancers with complicated volumes and/or close to radiosensitive organs while minimizing normal tissue complications and dose to organs at risk.

The excellent dose distribution is the utmost asset of proton beam therapy compared to photon-based modalities, with relative biological effectiveness (RBE) values slightly higher than 1 (1.1–1.4) [1]. For many tumor types, this can reduce radiation-induced long- and short-term side effects in the patients. This is important, especially in reducing the risks of secondary cancer in pediatric cancer treatment.

The most critical target for radiation-induced damage inside a cell is DNA. Ionizing radiation (IR) is known to cause many different lesions in the DNA molecule: single-strand breaks (SSB), double-strand breaks (DSB), base oxidations, abasic sites and DNA protein crosslinks [2]. When these lesions are not randomly distributed along the DNA helix within the cell nucleus volume but are located within an area of 10–20 base pairs of DNA length (few nm), it is defined as complex or clustered DNA damage, which is more complicated and difficult to repair than SSB. These clustered lesions are considered to be responsible for cell death and mutations [3,4]. The DNA repair efficiency in such cases depends on the location, types and number of lesions accumulated in close proximity [5,6]. Although clustered damages are linked to higher cell lethality and mutagenic potential [7], the stochastic formation perplexes its investigation.

Results in experimental radiobiology underline the direct relationship between clustered DNA damage and the interactions of IR with biological matter, especially the ionization cascades created along the tracks inside the target. Linear Energy Transfer (LET)-oriented studies shows, in most cases, an increased amount of clustered damage with increasing LET, leading to increased DNA fragmentation [8,9,10,11,12,13]. Monte Carlo simulations and multiscale mathematical approaches also agree with these results and suggest that LET (or, more exactly, the ionization density) plays a major role in determining the biological effects [6,14,15,16]. Therefore, there is a great need for DNA damage biomarkers for a better understanding of the normal tissue damage in proton and particle therapy beams and a deeper understanding of the mechanisms leading to either cell death or other effects [17]. RBE greater than unity is generally accepted to reflect the increased complexity of DNA damage induced by charged particles [1,18]. Several published studies underline the importance of DNA damage experiments using a variety of biological systems (plasmids, mammalian DNA and cells) to help us better understand the intriguing biological responses triggered by particles like protons or carbons [19].

The energy of the IR can either be deposited on the DNA macromolecule or the aqueous environment that surrounds DNA. As a result, DNA damage may occur either due to direct ionization or excitation of the molecule or due to indirect interaction with energetic electrons and products like Reactive Oxygen Species (ROS), created by radiolysis of water, which in their turn react directly/indirectly with the DNA [20,21]. This oxidative environment enhances the probability of induction of non-DSB clusters, often called OCDLs (Oxidative Clustered DNA Lesions) [22]. By adding scavengers in the aqueous environment before irradiation, the production of some of the reactants is eliminated, blocking the subsequent chain reaction and deactivating ROS. The type and the induction rate of DNA damage also depend on the free radical generation, scavenger concentration and DNA characteristics [20,23]. In the presence of radiation, the probability of radiolysis is quite high since water is the most abundant cell component.

The present work is a bottom-up approach to studying DNA damage, primarily induced by proton therapy beams, by employing a system of plasmid DNA in an aqueous solution. This is a simplified model, excluding cell response factors and when irradiated, it becomes a radiation damage detector. DNA molecules of full and known length in supercoiled (SC) form are vitiated by irradiation to a different extent according to radiation parameters and turn to circular (C), linear (L) and fragmented (F) forms. These forms can be detected by rapid electrophoretic methods, easily quantified and translated into a number of strand breaks. On the contrary, the number of short fragments of DNA is more arduous to define and leads to an apparent complex damage decrease, probably due to underestimation. The number of short fragments can either be estimated through mathematical models or by employing more radical molecule visualization methods. The first method used in this study was Agarose Gel Electrophoresis (AGE) combined with the Cowan Model, a “traditional” estimation created to describe plasmid forms transitions and DNA breaks formation, while a second supplementary method used Atomic Force Microscopy (AFM), which could be a powerful but demanding method to detect fragments down to 20 nm long (60 bps) [24,25,26].

Indirect damage through free radicals has been investigated by the addition of Tris and C3CA ROS-scavengers in the initial plasmid solution before irradiation as a radioprotectant. Tris is a common buffer scavenging solution that generally protects from DNA denaturation and free radical attack. C3CA is a coumarin derivative, and coumarins show high biological activity and low toxicity and are commonly used components in cancer treatment (prostate and renal cancer and leukemia) since they have the ability to counteract the side effects of radiotherapy [27].

## 2. Results and Discussion

### 2.1. AGE

**Figure 1:**
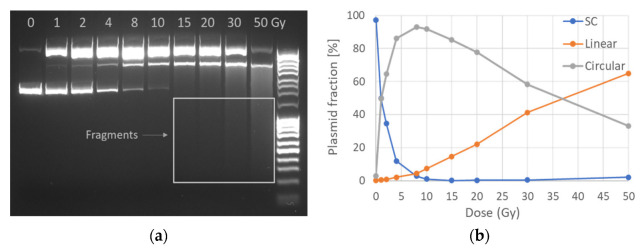
Electrophoretic plasmid analysis: (**a**) gel image of plasmid form transition for 10 different doses from 0 to 50 Gy with DNA ladder also included (right column). The original image has been slightly enhanced by increasing intensity in order to reveal low-intensity bands (small fragments) but not altered in other editing ways; SC, C and L are separated as expected due to their different DNA molecule mobility in the field. The image presented was oversaturated in order to visualize the fragments (smear in the last lanes). (**b**) Three plasmid forms transition with increasing dose as quantified according to gel band intensity.

The DNA electrophoresis adaptation in this study reveals an easily identified transition between SC, C and L forms of DNA which follows the dose increment and increased fragmentation (Figure 1a). In general, one expects that as the dose increases, the intact SC form is dramatically reduced, i.e., within the first 10 Gy. Of course, this phenomenon is also dependent on the scavenging capacity of the solution, plasmid type and water content, radiation quality and energies of the particles [28]. Simultaneously, the increasing dose of IR produces more breaks recorded as C and L bands (top and middle bands equivalently inside the gel). C is dominant for the first 10 Gy, while the linear DNA production rate (DSB formation) increases after the saturation point of the circular (Figure 1b). For doses higher than 10 Gy, the presence of the SC form is less than 1% and what we detect is the further breakage of C and L plasmid, producing gradually smaller fragments, leading to the formation of smear in the Agarose Gel Electrophoresis (AGE) gels.

**Figure 2:**
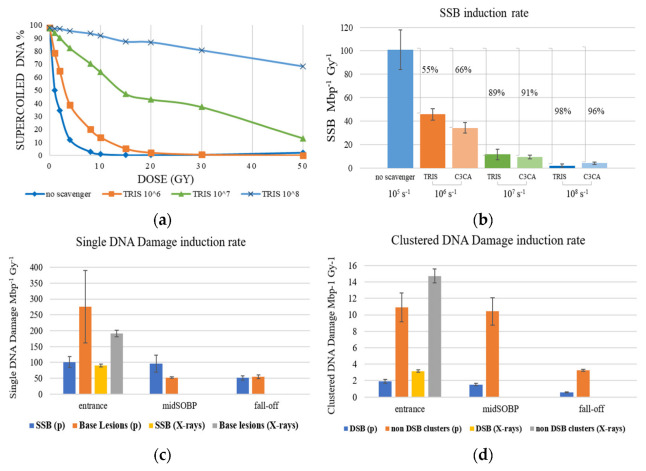
Proton (p) and X−rays−induced DNA damage. (**a**) Proton (entrance)−induced damage revealed as the decrease of SC plasmid with dose for Tris scavenging capacity ranging from no scavenging (10^5^ s^−1^ of residual Tris) to 10^8^ s^−1^, revealing the radioprotectant shielding of the plasmid integrity (up to 90%) the highest Tris concentration. (**b**) SSB Mbp^−1^ Gy^−1^ versus scavenging capacity for Tris and C3CA for 198 MeV protons at the entrance of the beam proves concentration-dependent manner. (**c**) Average number of SSB and single base lesions produced along the Proton Treatment Plan or X−rays, respectively. (**d**) Average number of DSB and non−DSB clustered damage produced along the Proton Treatment Plan or X−rays. The positions are the entrance of the beam, the middle of the SOBP plateau (midSOBP) and SOBP fall-off, as seen in Figure 3 and Table 1.

The above pattern is also followed for samples containing the ROS radioprotectants (scavengers) Tris and C3CA. As shown in Figure 2, an increase in scavengers’ concentration results in an overall decrease in SSB formation (which is mainly due to ROS attack). Figure 2a presents proton (entrance)-induced damage detected as the decrease of SC fraction with a dose for different Tris scavenging capacities, from no scavenging (10^5^ s^−1^ of residual Tris) to 10^8^ s^−1^, revealing radioprotection of the plasmid integrity up to 90%. This also proves that a significant amount of the DNA damage is not caused by the initial particle-target interactions, as the most amount of breaks on SC is caused by water radiolysis products. In Figure 2b, the SSB Mbp^−1^ Gy^−1^ versus scavenging capacity for Tris and C3CA for protons 198 MeV at the entrance of the beam proves that both scavengers protect DNA from ROS-mediated fragmentation in a concentration-dependent manner from 55% to 98%.

**Figure 3:**
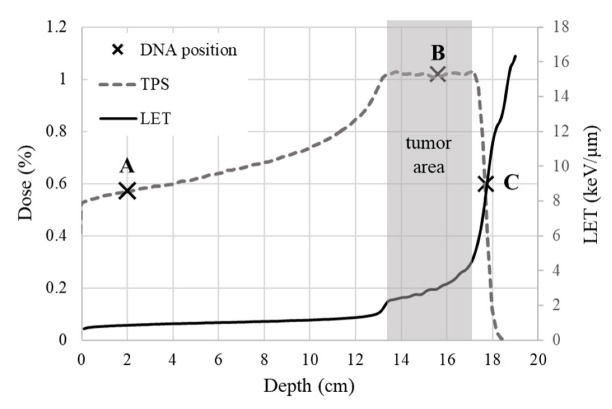
General treatment plan dose distribution: the % dose (dashed line) deposited when irradiating a volume of a hypothetical tumor of 4 cm thickness and approximately 13 cm under the skin (grey tumor area), as exported by TPS and the equivalent LET change over depth in water (solid line). The three positions of DNA irradiation (×) along the treatment plan are also marked: the beam entrance (A), the middle of SOBP plateau (B) and the SOBP fall-off (C).

The different positions along the treatment plan (Figure 3), and the subsequent relatively slight difference in LET values (Table 1), are also presented in Figure 2. Both strand breaks and base lesions (breaks deliberately created during post-irradiation treatment by restriction enzymes on sites of oxidized purines and pyrimidines) for protons and X-rays are grouped in SSB (Figure 2c) and DSB (Figure 2d) histograms. SSB is accompanied by a number of single base lesions (Figure 2c) that are 2.7 and 1.1 times the SSB value at beam entrance and SOBP fall-off. This no remarkably different pattern recorded in SSB along the beamline is overturned at the middle of the SOBP position, with base lesions being 0.5 times the SSB. Furthermore, SSB is 29–88 times more than DSB in each case, with the difference between entrance and SOBP within the error range for both SSB and DSB, probably due to similar LET values. Specifically, the mean rates of damage induction at the entrance of the proton beam are:

101.101 ± 16.868 SSB Mbp^−1^Gy^−1^, 1.914 ± 0.247 DSB Mbp^−1^Gy^−1^, 275.852 ± 114.295 Base Lesions Mbp^−1^Gy^−1^, 10.908 ± 1.778 non-DSB Clusters Mbp^−1^Gy^−1^, and for the irradiation with X-rays at the same position we record 89.559 ± 4.490 SSB Mbp^−1^Gy^−1^, 3.147 ± 0.141 DSB Mbp^−1^Gy^−1^, 191.102 ± 10.867 Base Lesions Mbp^−1^Gy^−1^, 14.723 ± 0.856 non-DSB Clusters Mbp^−1^Gy^−1^. Figure 2d shows that clustered DNA damages produced by X-rays outnumber those created by proton beams at the entrance. This may originate from the very low LET for the protons in the entrance that is similar to that of X-rays (~1 keV/µm); therefore, no major differences are expected in general. Furthermore, the contribution of non-DSB oxidative damages in the clusters measured in the case of X-rays is expected to be high, translated into higher levels of clustered damage. Interestingly enough, previous studies [29,30,31] support the prevalence of oxidized base damages in the case of low-LET radiations such as X-rays. Considering that a percentage of these non-DSB lesions may almost instantly be converted to DSBs through the lyase/endonuclease enzymatic activity used in the present assays, then this may lead to higher levels of clusters as measured in the electrophoresis. Although clustering induced by X-rays appears to numerically exceed clustering produced by protons at the entrance of the beam, proton radiation is more efficient than X-rays, as evidenced by the ratio of non-DSB clusters to DSB for X-rays (4.7) being higher than that for protons (5.7).

At the middle of the SOBP plateau, the mean damage induction rate recorded is 96.012 ± 27.211 SSB Mbp^−1^Gy^−1^, 1.529 ± 0.169 DSB Mbp^−1^Gy^−1^, 52.085 ± 2.365 Base Lesions Mbp^−1^Gy^−1^, 10.415 ± 1.666 non-DSB Clusters Mbp^−1^Gy^−1^. At the fall-off of SOBP, where we expect to have higher LET values, the recorded damages were not greater as estimated by simulations for increased LET values but were not always verified by experimental studies [9]. This may have two interpretations. Considering the steep slope shift in LET and the dose distribution at the end of the beam trajectory (Figure 3), where LET increases abruptly but not critically, lower levels of DNA damage is an astounding proof of the successful use of protons for patient treatment since the healthy tissue area in very close proximity (within a few millimeters) of the tumor is only mildly stressed. Especially the low DSB and clustered damage levels, which are potentially lethal for a cell, imply a relatively safe cell environment. On the other hand, the apparent lower level of DSB and clustered damage recorded at the SOBP fall-off might be a result of underestimation of the damage due to the highly complex and fragmented DNA molecules, as also arises by AFM results. This is probably due to the limitations of the method since, in agarose gel electrophoresis, shorter DNA fragments may escape due to their higher mobility. The values of the mean damage induction rate recorded at the SOBP fall-off are 51.069 ± 6.923 SSB Mbp^−1^Gy^−1^, 0.579 ± 0.060 DSB Mbp^−1^Gy^−1^, 54.453 ± 6.350 Base Lesions Mbp^−1^Gy^−1^, 3.255 ± 0.092 non-DSB Clusters Mbp^−1^Gy^−1^.

**Figure 4:**
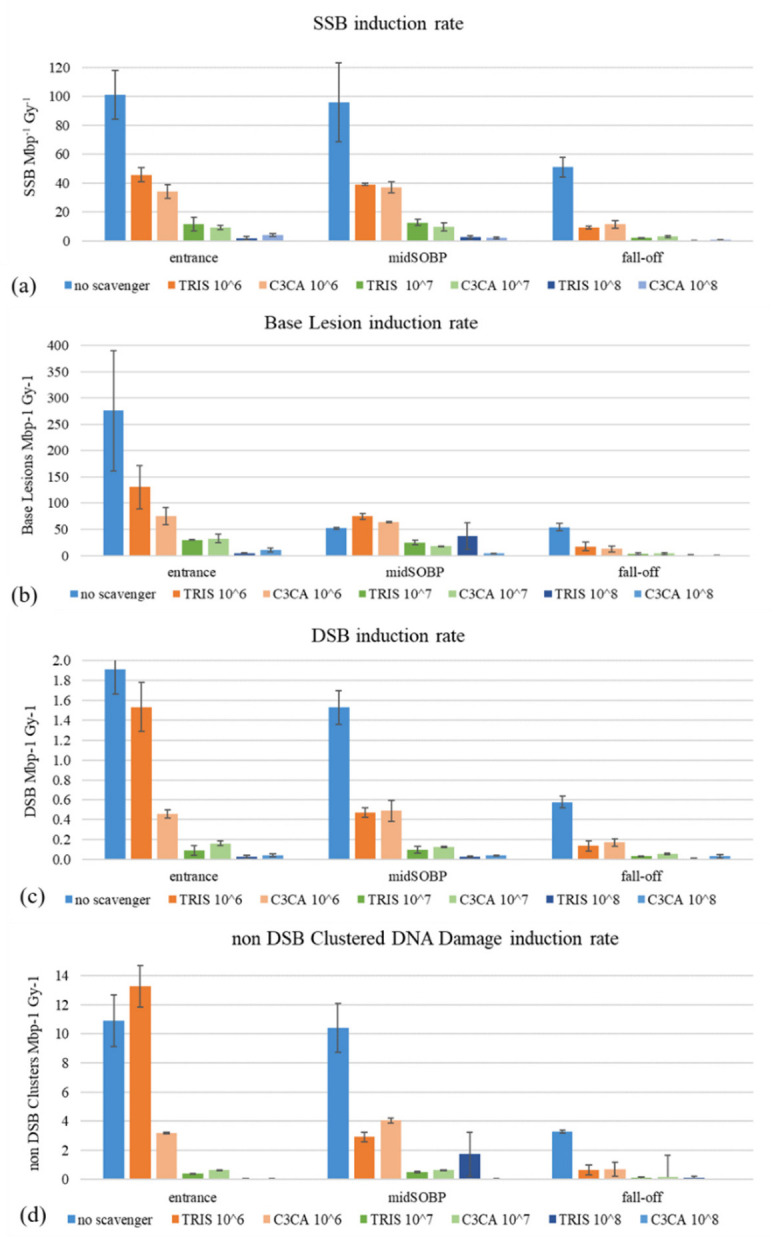
Proton-induced DNA damage: (**a**) Average number of SSB (SSB Mbp^−1^ Gy^−1^), (**b**) DSB (DSB Mbp^−1^ Gy^−1^), (**c**) Base Lesion and (**d**) non-DSB Clustered DNA damage induction rate versus increasing the scavenging capacity of Tris and C3CA scavengers (from 10^−6^ to 10^−8^ s^−1^) for the three irradiation positions: the entrance of the beam (entrance), the middle of the SOBP plateau (midSOBP) and SOBP fall-off (fall-off).

Experimental results for different scavenging conditions are presented in Figure 4 and for all irradiation positions along the Proton Treatment Plan: proton beam entrance (LET = 1 keV/μm), middle of SOBP plateau (LET = 3 keV/μm) and SOBP fall-off (LET = 9 keV/μm), for Tris and C3CA radioprotectants in all three different values of scavenging capacity (10^6^ s^−1^, 10^7^ s^−1^, 10^8^ s^−1^) and 10^5^ s^−1^ of residual TRIS (no scavenger). Overall, we detect a decrease in the induction of proton-induced DNA damage with increasing scavenging capacity of the solutions. Comparing reduction in damage levels, the general trend is that with increasing LET (from the entrance to mid-SOBP and fall-off), the dependency on the antioxidant concentration is reduced.

**Figure 5:**
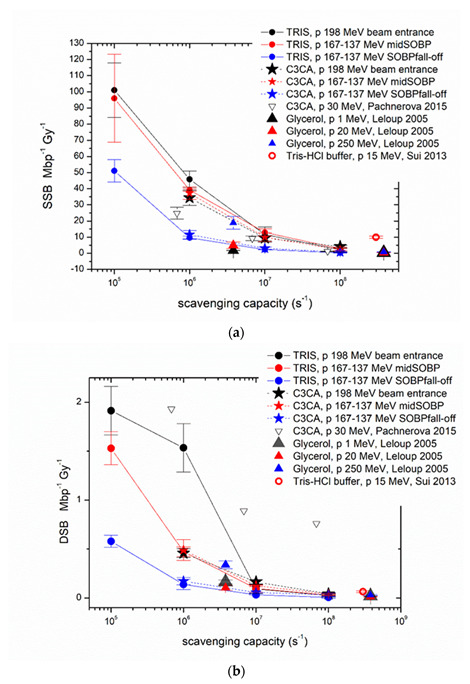

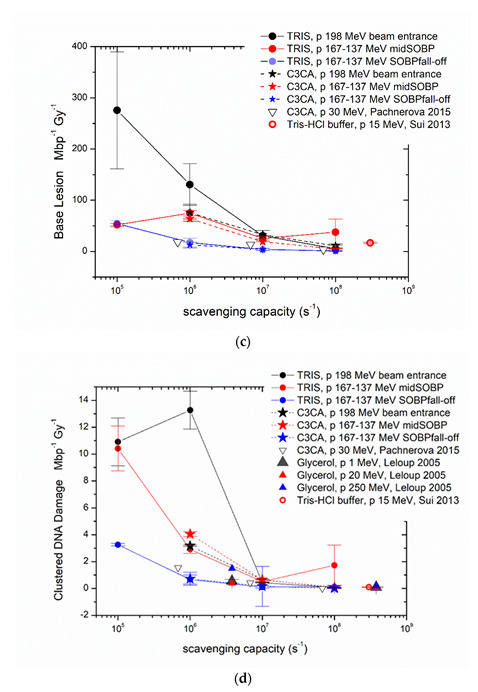
Proton (p)−induced damage yield (Mbp^-1^ Gy^-1^) versus scavenging capacity (s^−1^) as calculated in the present study for Tris and C3CA (lines are used as guides to the eyes) and literature values from studies using plasmid models with comparable proton energies and solution scavenging capacity. (**a**) SSB, (**b**) DSB, (**c**) Base Lesion, (**d**) Clustered DNA damage induction rates result from a variety of irradiation conditions, e.g., plasmid in solution or dried, different plasmid concentration and proton energy, details that can be found in Table A1. (see Refs. [28,32,33,34]).

Figure 5a–d shows our experimental values together with the comparable literature data on the plasmid model. The numerical values of the present radiation conditions and the comparable literature data of the plasmid model can be found in Table A1 (Appendix A), accompanied by all critical radiation and sample parameters. For example, for DSBs in, the entrance reduction between ‘no scavenger’ to C3CA is ~76% falling to ~65% for the SOBP. Similarly, for non-DSB (base) clusters, from ~75% reduction (entrance), we go to ~60% for the SOBP region.

In Figure 5, we have included cumulative data from different studies using protons of different energies and under different scavenging capacities. Closed circles correspond to the data from the present study, which are in good agreement with the literature data. One general comment is that when reviewing the data from other studies (Table A1), it can be seen that there is a great variety of values and, in some cases, disharmony between our experimental output and the others’ data. Of course, as already explained above, these type of irradiation experiments depends on several physical and chemical parameters that may change the overall interaction of protons with DNA. A comparison with results within the scavenging range of the present study (Figure 5a–d) confirms the strong DNA damages elimination by scavengers depicted in Figure 4a–d. Although it is generally believed that 60–70% of the biological effects of low LET IR in mammalian cells are caused by indirect action [35,36], plasmid studies show larger proportions of indirect damage through more efficient scavenging. Plasmid studies testing scavenging high LET carbon ion-induced damage also report results implying 96% of the total SSB amount is attributed to indirect damage [37,38]. The aqueous nature of the plasmid solutions probably enhances such findings due to the high radiolysis probability.

By using the mean rate of DSB induction for protons and dividing by the DSB induction rate for X-rays at the entrance of the beam, we estimated an apparent RBE value of 0.61 at the entrance, 0.49 in the middle of the SOBP plateau and 0.18 at the SOBP fall-off. The reported [39] average RBE values for cell survival are 1.1 at the entrance, 1.15 in the center, 1.35 at the distal edge and up to 1.7 in the distal fall-off of the SOBP. These values are calculated with clonogenic cell survival as an endpoint. It is generally accepted that endpoints other than clonogenic survival produce such diverse results that do not allow to avoid the general statement that the RBE is, on average, in line with a value of ~1.1 [40]. RBE values of the present study are based on a simple DNA system of plasmid in an aqueous solution, which excludes the cell response and their repair mechanisms. This model serves well when the focus is on damage (excluding cell response and repair), and the presented RBE values should be considered as damage-calculated. Comparison with the literature values from cell survival would be unfair, first due to the different complexity of the systems and calculation methods, but also due to the fact that the generally accepted RBE values are under question, given the diversity of the results from different irradiation conditions like cell lines, dose and LET.

### 2.2. AFM

**Figure 6:**
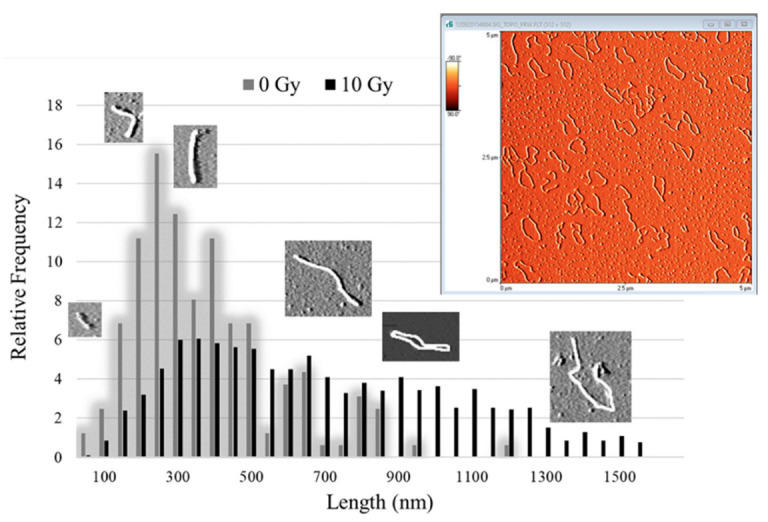
Atomic Force Microscopy (AFM) measurements: The relative frequency distribution of apparent molecule lengths for control sample (0 Gy) and irradiated with 10 Gy (proton beams) together with AFM images of DNA molecules of different forms. A representative AFM image (5 μm × 5 μm) of plasmid pBR322 on freshly cleaved mica surface, revealing the DNA macromolecule conformations. Such images are used as input for length evaluation and spread-out full-length circular molecules are utilized as calibration rulers for pixel-to-nm conversion. The length of the super-twisted supercoiled fraction is underestimated due to its adsorption on the mica surface. As a result, the apparent length of control plasmid is recorded smaller, while plasmid irradiated with plasmid is distributed along the whole range of lengths.

**Figure A1:**
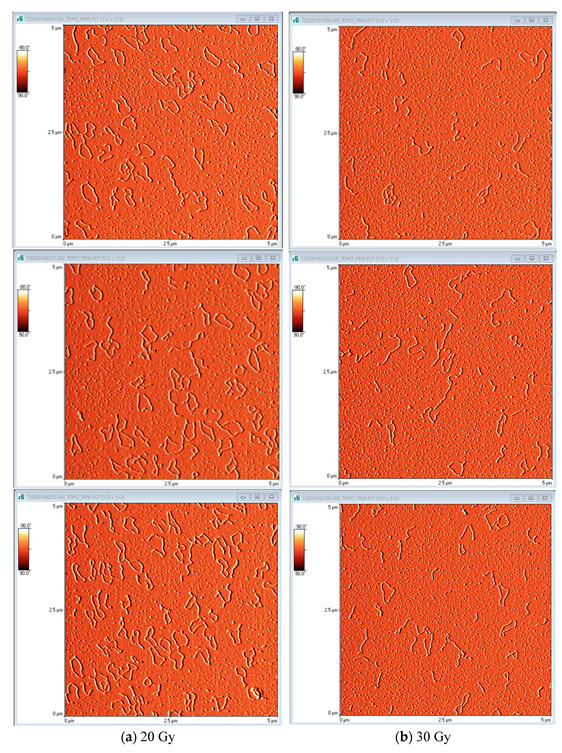
AFM topographic images of plasmid pBR322 on mica substrate irradiated with protons: (**a**) 20 Gy and (**b**) 30 Gy. AFM reveals the different conformations of DNA from simple linear to complex twisted loops. The difference in plasmid concentration between the two doses is incidental.

The results from the AFM length measurements are organized into histograms presenting length distributions in 50-nm-wide bins in the range of 0 to 1550 nm. The relative frequency of calibrated-from-pixel-to-length plasmid sizes was chosen to present and evaluate, and not show the apparent length, because of the variance in the number of evaluable molecules (N) on mica surfaces for each experimental condition. Figure 6 presents a typical topographic AFM image of plasmid on mica (see more in Figure A1 in Appendix A) and the relative frequency distribution of the lengths of 0 Gy (control) and 10 Gy. The 0 Gy distribution shows the detection of tight-binding DNA conformations of total length mostly of ~200–300 nm, underestimating the length of the supercoiled fraction due to the adsorption of the 3D molecules on the 2D mica surface. On the other hand, 10 Gy distribution reveals the segmentation of the initially supercoiled plasmid into one-stranded unfolded DNA pieces by recording molecules along the whole distribution range.

**Figure 7:**
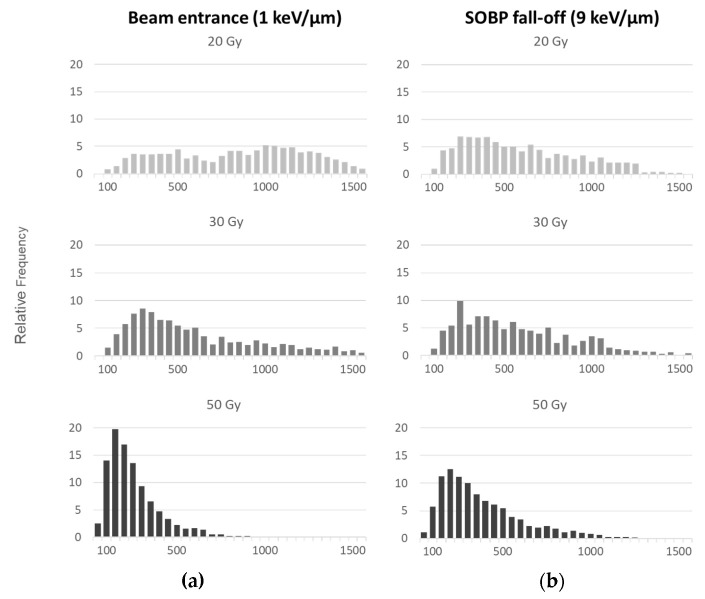
Relative frequency distributions of apparent molecule lengths measured using AFM for plasmid pBR322 irradiated with doses of 20, 30 and 50 Gy at (**a**) the entrance of proton beam (LET = 1 keV/μm) and (**b**) SOBP fall-off (LET = 9 keV/μm).

With increasing dose (Figure 7), there is a shift to smaller plasmid lengths revealed as increased DNA fragmentation due to multiple breaks. The same trend seems to follow the LET increase, since even in the case of small increment as in our experiment (Figure 7a corresponds to proton beam entrance and LET = 1 keV/μm, while Figure 7b to SOBP fall-off plasmid position with LET = 9 keV/μm) the fragment distribution shifts to smaller linear recorded DNA forms.

## 3. Materials and Methods

### 3.1. Sample Preparation

Plasmid pBR322, a vector of 4361 base pairs, dissolved in 10 mM Tris–Hcl, 1 mM EDTA (New England BioLabs Inc., Ipswich, MA, USA), was purified from salts via dialysis in ultrapure water (Pur-A-Lyzer dialysis kit from Sigma-Aldrich, St. Louis, MO, USA). Our study included two types of scavengers: coumarin-3-carboxylic acid (C3CA) (a water-soluble coumarin derivative) and 2-amino-2-(hydroxymethyl)propane-1,3-diol (Tris) with hydroxyl radicals (•OH) reaction rate constant k_C3CA_ = 6.8 × 10^9^ M^−1^ s^−1^ [41] and k_Tris_ = 1.5 × 10^9^ M^−1^ s^−1^ [42]. Three forms of plasmid samples were prepared: plasmid without scavenger, plasmid with C3CA and plasmid with Tris in total scavenging capacity of 10^6^ s^−1^,10^7^ s^−1^ and 10^8^ s^−1^ for each compound. Scavenging capacity equals the product k × S, where S is the scavenger concentration. Every sample contained 10 ng/μL of the plasmid in 20 mM potassium phosphate buffer, pH 7, ultrapure water and scavenger solution in a total volume of 160 μL irradiated in a polypropylene microtube (0.5 mL) with 0.85-mm-thick walls.

### 3.2. Irradiation and Dosimetry

Samples were irradiated at MedAustron Ion Beam Therapy Center in Wiener Neustadt, Austria, with doses up to 50 Gy at room temperature and kept on ice before and after irradiation.

#### 3.2.1. Set-Up

The set-up that held all sample tubes consisted of a Teflon frame holder that allowed positioning of a removable plastic base at different heights. This frame was always placed vertically in the metal base of the solid water RW3 slab phantom, which was also used for a standard dosimetric quality assurance procedure by means of UNIDOSwebline with the ionization chamber ROOS (PTW, Freiburg, Germany).

#### 3.2.2. Proton Irradiation

Treatment planning and dose calculations were performed with the RayStation TPS V5.99 (RaySearch, Stockholm, Sweden) for a hypothetical scenario of an existing tumor, 4 cm thick and 13 cm behind the skin. The set-up was placed on a robotic table (KUKA robot, Reutlingen BEC GmbH, Pfullingen, Germany), and then the table was placed in front of the beam, in a position that was calculated during the treatment planning. The proton beams were of energy range 137–198 MeV with the corresponding LET values 1–10 keV/μm along the clinical SOBP (Figure 3), a LET range that is important to cell killing [43]. The samples were irradiated in order of minutes under room temperature, and they were otherwise kept on ice. Doses of 0, 1, 2, 4, 8, 10, 15, 20, 30 and 50 Gy were delivered in different positions along the proton treatment plan, and an ionization chamber was used for dose monitoring. The chosen positions were achieved by interfering with polymethyl methacrylate (PMMA) plates of calculated thicknesses in front of the samples (Table 1).

#### 3.2.3. X-rays

A YXLON X-ray unit (Yxlon International X-ray GmbH, Hamburg, Germany) was employed for plasmid irradiation. The X-ray tube was adjusted to 200 kV, 20 mA, and the dose rate was found to be constant at 1.25 Gy/min. Since the source tube was horizontal and the ROOS chamber set-up prerequisites 1 mm PMMA plate to position the plane parallel chamber vertically to the irradiation axis, all samples were exposed and taped on the back side of that PMMA plate and kept on ice before and after irradiation.

### 3.3. Post-Irradiation Treatment—DNA Damage Detection

#### 3.3.1. Agarose Gel Electrophoresis in Tris-Acetate-EDTA (TAE) Buffer

Three subsamples of 5 μL, derived from each sample, were incubated at 37 °C for 1 h without or with enzymes: Formamidopyrimidine DNA glycosylase (Fpg) or Endonuclease III (Nth) in specific complementary reaction buffers (New England BioLabs Inc.). Both enzymes are *E. coli* base excision repair enzymes and catalyze cleavage of oxidized nitrogenous bases. Fpg catalyzes lesions in purines, while Nth in pyrimidines and, due to associated lyase activity, they convert both existing and resulting abasic sites and oxidized bases into DNA strand breaks [44]. The optimal enzyme concentration was found by titration of each enzyme to succeed maximum specific along with minimum non-specific cutting activity and by comparing irradiated with non-irradiated samples treated with different enzyme concentrations. Enzymatic reactions were quenched by adding 2 μL of DNA loading dye (MassRuler, ThermoFisher, Waltham, MA, USA) containing bromophenol blue, 3.3 mM Tris–HCl and 11 mM EDTA. The different forms of plasmid DNA were separated and then visualized by employing agarose gel electrophoresis on 1% agarose gel stained with fluorescent dye SYBR Green I (Sigma-Aldrich) under electric field of 100 V in 0.5 × TAE.

**Figure 8:**
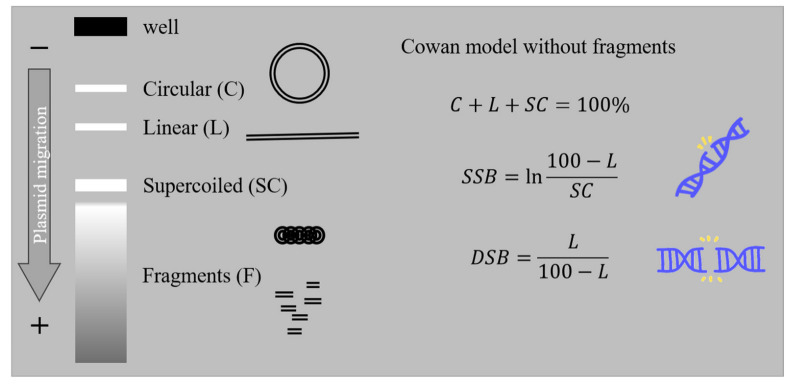
The electrophoresis concept: Each plasmid sample is added in a well (cathode side), electric field is applied at the gel edges and under the electric potential difference, the negatively charged DNA migrates to the anode side through the gel net. Due to the net-like agarose gel, different DNA forms are separated according to the size of each form that created three clearly distinguishable bands. Supercoiled (SC) plasmid (small-sized twisted DNA) is moving easier and faster, crossing a larger pathway inside the gel and appearing as a more distant band. The large circular (C) and linear (L) molecules have lower mobility than SC, and they are trapped in areas closer to the well. Therefore, the unfolded C appears as higher band in the gel, and L forms a band in between. Additionally, smaller DNA fragments (F) of higher mobility appear as a smear inside the gel. SC is the compact initial DNA with no break; C is caused by the cleavage of one DNA strand (SSB) and L by the cleavage of two strands (DSB). F results from further nicking cannot be safely measured via AGE and is excluded from present analysis. By defining the amount of SC, C and L and employing Cowan model [45] (equations above), we translate it into number of SSB and DSB.

At this stage and under the presence of single- and double-strand breaks, plasmid DNA is separated into bands-fractions of different conformations: supercoiled (SC), circular (C) and linear (L) and fragments (F), with the last one being non-detectable due to method limitations (Figure 8). These patterns were visualized with green filter on an MYECL Imager, and band analysis was performed with the image processing program MYImageAnalysis™ v2.0 Software (Thermo Scientific, Waltham, MA, USA). The full-length molecule bands of SC, R and L were identified and integrated. The attempt to include F in the analysis was not always possible, as shorter-than-linear plasmid lengths have greater mobility in the electric field, appearing as a smear under L and SC bands that were not always countable. Averaged SC, C and L relative fractions are quantified by gel luminosity and translated into DNA breaks using Cowan model [45,46] (Equations in Figure 8).

The slope of SSB and DSB vs. dose plots converts results into the useful quantity of G-values (breaks per Gy per Mbp). To improve the statistics, AGE was performed twice for each irradiation condition, and image analysis was performed twice for each gel. The irradiation was only repeated for the middle of SOBP. Results are the mean values accompanied by the error of the mean.

Base lesions and non-DSB clustered damage are calculated via the difference between enzymatic cleavage and strand breaks. Base lesions correspond to the excessive SSB, and non-DSB clustered damage is the excessive DSB that is produced by Nth and Fpg enzymes.

#### 3.3.2. AFM Analysis

Samples were also analyzed with an atomic force microscope. A part of the dialyzed (or/and also irradiated) plasmid sample was diluted to 1 ng/μL pBR322 in 1 mM MgCl_2_. Mg^2+^ cations of such a concentration stabilize double-stranded DNA, prevents complete denaturation of the DNA and enhances adsorption (via a weak electrostatic attachment) of the molecule onto mica substrate [28,47]. AFM images (Figure A1) were acquired with Nanoscope diInnova device (Veeco Metrology, Santa Barbara, CA, USA) with an Innova scanner possessing a maximum range of 100 μm × 100 μm in tapping mode. Antimony (n) doped silicon tips with a nominal constant 3 N/m and 42 N/m (Bruker, Billerica, MA, USA) were used to acquire several high-resolution DNA topographies of 5 × 5 μm^2^ at a scanning speed 1 Hz and with lateral resolution 512 × 512 pixels. The images were processed with the AFM apparatus software (SPMLab 5.01, Sunnyvale, CA, USA), they were flattened, and a histogram equalization was applied for the background noise subtraction and image contrast increase.

Images were input into the semi-automatic algorithm “lemeDNA” (Length Measurement of DNA) [48]. This algorithm uses 8-connected Freeman chain code to compute the pixel lengths and estimate the length of each molecule in pixels. Since the length of an intact plasmid pBR322 molecule is known to be 4361 base pairs, with each base pair 0.34 nm long (X-ray crystallography studies), calibration from pixels to nm is feasible and arbitrary fragments of DNA can be organized in length distributions.

## 4. Conclusions

The damage induction rate that follows the increasing capacity was found to decrease, underlining dominant indirect effects and the leading role of ROS in proton irradiation treatment. Enzyme analysis reveals that base lesions and non-DSB clusters are increased compared to SSB and DSB, respectively, with base lesions being the leading type of damage. Different positioning along the proton treatment plan shows no remarkable difference between DNA damage recorded at the beam entrance and at the middle of the SOBP position, probably due to very similar LET values. The fact that there is no higher damage induction rate at the distal fall-off of SOBP, as hypothesized for increasing LET, is raising the question about possible underestimation of highly complex damage and if there is multifragmented DNA that is not detectable with AGE and/or other types of interactions between DNA and protons that we do not fully comprehend.

Comparison with the literature suggests a great variety of results since one finds experiments with different beam energy, irradiation conditions and set-ups. Furthermore, although there are studies that invoke the plasmid model, the systems are finally different because there is strong dependence on plasmid type and geometry, scavenging capacity and plasmid hydration level. All these parameters blend together, complicating result interpretation.

AFM provides impressive images of DNA conformations and visualizes fragments as short as 42 bp (equal to 14 nm). The present analysis with the LemeDNA algorithm qualitatively records the shift of the relative frequency distribution of the apparent DNA length towards shorter lengths following the dose increment. On the other hand, quantitatively, there is not enough to contribute since there is no discrimination between different DNA forms, and only the apparent size is recorded. In order to reproduce results comparable to that of AGE, there is a need for an algorithm that also distinguishes and counts the SC and C and separates the L conformations into categories according to their measured length. This way, users will have the option to perform a similar but stronger analysis than this of traditional AGE.

Proton therapy-induced DNA damage in vitro studies employing biological models like plasmid DNA or cells using real therapeutic beams and exposure conditions are always challenging and quite limited. The cost of proton irradiations is high, and irradiation time is distributed on a priority basis, with patients being the first concern, with clinical research and applied and basic research activities following. At the same time, each ion facility is developed using different technologies (beam physical characteristics, dose delivery system, irradiation set-up, etc.), producing results that are not always repeatable from another beam facility. Therefore, non-clinical studies are difficult to perform and repeat in ion therapy facilities, with the available proton DNA damage data sourcing from the not plentiful literature of different parameters.

Consequently, the present study provides results from experiments performed in a cutting-edge ion therapy facility and analyzed with updated methods. The current results constitute a fresh input for early physical-chemical events of DNA damage induction. At the same time, this work strengthens the challenging attempt toward the modeling and deeper understanding of IR therapeutic modalities’ effects on biological systems that may discharge research from demanding experiments in the future. Last but not least, DNA-based systems have been recently used as reliable dosimeters in therapy beam set-up. More specifically, recent independent studies on DNA double-strand breaks measurement were performed using a DNA dosimeter, consisting of magnetic streptavidin beads attached to a properly labeled four kbp DNA molecule suspended in phosphate-buffered saline (PBS) and utilized as a method of radiation measurements for therapeutic beams and high doses > 25 Gy where cellular systems are very difficult to be utilized [49]. Based on all the above, our results may also prove useful in the development of more accurate bio-dosimetry in proton or carbon-therapy treatment planning incorporating the biological signature of radiation and aid reduce damage to normal tissues and toxicity.

## Figures and Tables

**Table 1:** Irradiation parameters along the therapeutic proton SOBP.

Irradiation Position	Depth in PMMA (cm)	LET (keV/μm)	Energy Range (MeV)
A—beam entrance	2	1	198
B—plateau of SOBP	15	3	137–167
C—tail of SOBP	16.8	9	137–167

## Data Availability

All data will be provided by corresponding authors upon logical request.

## Appendix A

**Table A1:** IR (protons, ions and electromagnetic radiation) damage induction rate on plasmid systems: results from the present study and reported in the literature.

	Type Rad/Proton Energy (MeV)	LET (keV/μm)	Scavenger	Plasmid	Plasmid Concentration (ng/μL)	Scav. Capacity (s^−1^)	SSB Mbp^−1^ Gy^−1^	Error	DSB Mbp^−1^Gy^−1^	Error	Base Lesions Mbp^−1^ Gy^−1^	Error	Non−DSB Clusters Mbp^−1^ Gy^−1^	Error
this study	X−rays		no scav (residual TRIS)	pBR322 in solution	10	10^5^	89.559	4.490	3.147	0.141	191.102	10.867	14.723	0.856
			TRIS			10^6^	30.232	1.094	0.532	0.215	65.596	3.163	3.919	0.836
						10^7^	4.551	0.746	0.103	0.010	18.656	3.415	0.631	0.036
						10^8^	1.135	0.300	0.108	0.077	4.654	1.005	0.263	0.026
			C3CA			10^6^	24.306	1.194	0.606	0.109	61.223	6.884	3.991	−0.054
						10^7^	4.921	0.108	0.117	0.011	9.562	0.111	0.726	0.037
						10^8^	1.370	0.054	0.121	0.077	2.139	0.133	0.137	0.032
this study	p 198 MeV	1	no scav (residual TRIS)	pBR322 in solution	10	10^5^	101.101	16.868	1.914	0.247	275.852	114.295	10.908	1.778
		1	TRIS			10^6^	45.848	5.031	1.534	0.247	130.510	40.667	13.270	1.411
		1				10^7^	11.609	4.668	0.092	0.047	30.405	−0.222	0.426	−0.015
		1				10^8^	2.083	1.355	0.025	0.015	5.103	0.227	0.078	−0.011
		1	C3CA			10^6^	34.349	4.683	0.458	0.044	75.715	16.431	3.175	−0.042
		1				10^7^	9.434	1.499	0.163	0.022	32.678	8.270	0.658	0.023
		1				10^8^	4.141	0.861	0.041	0.014	10.709	3.961	0.052	−0.013
	p 167−137 MeV	4	no scav (residual TRIS)	pBR322 in solution	10	10^5^	96.012	27.211	1.529	0.169	52.085	2.365	10.415	1.666
		4	TRIS			10^6^	39.171	0.754	0.473	0.048	74.808	5.202	2.912	0.305
		4				10^7^	12.888	2.234	0.096	0.035	25.294	4.359	0.509	0.039
		4				10^8^	2.727	1.152	0.028	0.005	37.634	25.479	1.727	1.516
		4	C3CA			10^6^	37.170	3.826	0.490	0.107	63.820	−1.111	4.053	0.178
		4				10^7^	9.928	2.765	0.125	0.009	18.926	0.070	0.642	0.003
		4				10^8^	2.220	0.786	0.039	0.006	4.596	0.446	0.081	0.005
	p 167−137 MeV	9	no scav (residual TRIS)	pBR322 in solution	10	10^5^	51.069	6.923	0.579	0.060	54.453	6.350	3.255	0.092
		9	TRIS			10^6^	9.581	0.945	0.138	0.052	17.400	8.311	0.671	0.338
		9				10^7^	2.274	0.235	0.032	0.001	3.745	1.864	0.106	0.085
		9				10^8^	0.299	0.089	0.005	0.009	1.269	0.619	0.115	0.121
		9	C3CA			10^6^	11.545	2.456	0.172	0.036	12.770	5.628	0.716	0.481
		9				10^7^	3.085	0.691	0.055	0.004	4.010	1.940	0.157	1.495
		9				10^8^	0.717	0.237	0.032	0.016	0.867	0.253	0.009	0.018
Pachnerová 2015 [32]	p 30 MeV	1.9	TE buffer	pBR322 in solution		1.5 × 10^3^	113.760	17.064	3.990		113.090		14.580	
			C3CA			6.8 × 10^5^	24.790	3.718	1.930		18.020		1.540	
			C3CA			6.8 × 10^6^	9.170	1.376	0.890		13.320		0.410	
			C3CA			6.8 × 10^7^	1.220	0.182	0.760		3.390		0.000	
Sui 2013 [33]	p 15 MeV	3.6	TRIS−HCl	pUC19 in solution	100	3 × 10^8^	9.950	0.790	0.064	0.009	16.560	1.400	0.100	0.016
Leloup 2005 [34]	p 249 MeV	0.39	glycerol	pHAZE liquid film		3.8 × 10^6^	18.850	3.900	0.338	0.039			1.495	0.260
	p 19.3 MeV	2.7				3.8 × 10^6^	5.005	1.950	0.104	0.026			0.377	0.163
	p 1.03 MeV	25.5				3.8 × 10^6^	1.690	0.260	0.163	0.020			0.533	0.130
	p 249 MeV	0.39	glycerol			3.8 × 10^8^	0.917	0.195	0.029	0.006			0.176	0.026
	p 19.3 MeV	2.7				3.8 × 10^8^	0.397	0.091	0.011	0.004			0.037	0.020
	p 1.03 MeV	25.5				3.8 × 10^8^	0.384	0.059	0.018	0.002			0.085	0.020
Vyšín 2015 [50]	p 10 MeV	6.39	TE buffer	pBR322 dry	30		0.009	0.007	0.003	0.001	0.082		0.001	
	p 20 MeV	3.64					0.053	0.000	0.001	0.001	0.088		0.006	
	p 30 MeV	2.61					0.044	0.006	0.001	0.000	0.088		0.004	
	p 20 MeV	3.1−6.95	TE buffer	pBR322 liquid			58.900	2.600	2.000	0.200	45.800		4.500	
	p 30 MeV	1.96−2.34					39.700	8.200	1.500	0.800	45.900		3.900	
Ohsawa 2021 [51]	p 27.5 MeV	2.3	TE buffer	pBR322 in solution	50		2.476	0.032	0.027	0.005				
	p 27.5 MeV FLASH	2.3					2.016	0.156	0.025	0.004				
Small 2021 [52]	e 100 MeV		no scav	pBR322 in solution			15.420	0.860	0.350	0.020				
	e 100 MeV FLASH						20.310	0.200	0.370	0.030				
	e 150 MeV						17.630	0.570	0.350	0.030				
	e 150 MeV FLASH						18.740	0.520	0.370	0.040				
	e 200 MeV						20.190	0.560	0.380	0.020				
	e 200 MeV FLASH						21.220	0.380	0.380	0.020				
	e 100 MeV	0.22		pBR322 dry			69.810	8.720	3.660	0.430				
	e 150 MeV	0.22					80.300	3.060	3.710	0.110				
	e 200 MeV	0.23					50.270	4.190	3.830	0.450				
Small 2019 [53]	60−Co − 6 MeV	0.19	no scav	pBR322			7.940	0.140	0.970	0.100				
	60−Co − 10 MeV	0.20					11.000	0.660	1.130	0.080				
	60−Co − 15 MeV	0.20					7.710	0.030	1.220	0.010				
Pachnerová 2019 [28]	Carbon ions 400 MeV/u	11	TE buffer	pBR322	10	1.5 × 10^3^	128.600	1.900	4.800	0.400				
Souici 2016 [54]	Ultra Soft X−rays 1.5 keV		TRIS	pBR322	5	1.5 × 10^6^	2.477	0.130	0.228	0.026				
						1.5 × 10^7^	0.780	0.059						
						1.5 × 10^8^	0.163	0.072						
					10	1.5 × 10^6^	2.386	0.052	0.208	0.020				
						1.5 × 10^7^	0.845	0.013						
						1.5 × 10^8^	0.182	0.020						
					50	1.5 × 10^6^	2.106	0.091	0.137	0.013				
						1.5 × 10^7^	0.800	0.039						
						1.5 × 10^8^	0.228	0.026						
Shiina 2013 [55]	X−rays		TRIS	pUC18 in solution	50	1 × 10^6^	5.720		0.111		9.035		0.481	
						1.5 × 10^7^	1.105		0.029		1.430		0.092	
						3 × 10^8^	0.163		0.008		0.650		0.020	
						1 × 10^10^	0.124		0.010					
	C6+ 290 MeV	13	TRIS	pUC18 in solution	50	1 × 10^6^	5.590		0.228		4.290		0.481	
						1.5 × 10^7^	1.300		0.008		2.795		0.182	
						3 × 10^8^	0.325		0.013		1.300		0.059	
Ushigome 2012 [56]	He2+	2.2		pUC18 hydrated films			0.058	0.013	0.005	0.001	0.141		0.004	
		6					0.064	0.012	0.004	0.001	0.169		0.007	
	C5+, C6+	13		pUC18 hydrated films			0.107	0.026	0.008	0.004	0.255		0.009	
		87					0.040	0.003	0.003	0.000	0.057		0.003	
		122					0.037	0.003	0.004	0.000	0.074		0.004	
		342					0.030	0.004	0.005	0.000	0.047		0.001	
		507					0.033	0.003	0.005	0.001	0.061		0.000	
	Ne10+	31		pUC18 hydrated films			0.092	0.023	0.005	0.001	0.180		0.006	
		361					0.038	0.003	0.007	0.001	0.066		0.002	
		491					0.031	0.001	0.007	0.001	0.067		0.002	
		842					0.031	0.005	0.008	0.001	0.038		0.002	
Urushibara 2008 [57]	gamma 60−Co			pUC18			0.047	0.003	0.005	0.000	0.138		0.014	
	α He	19		pUC18			0.045	0.007	0.002	0.001	0.166		0.006	
	α He	63					0.047	0.001	0.005	0.001	0.085		0.006	
	α He	95					0.044	0.002	0.007	0.000	0.047		0.004	
	α He	121					0.031	0.003	0.003	0.001	0.040		0.003	
	α He	148					0.025	0.002	0.006	0.000	0.012		0.002	
Yokoya 2003 [57]	α Pu						0.039	0.006	0.011	0.001	0.016		0.003	
Klimczak 1993 [58]	gamma 60−Co			pBR322	100	1.5 × 10^6^	26.00		0.26					
						1.5 × 10^7^	5.85		0.07					
						1.5 × 10^8^	1.30		0.02					
